# How COVID-19 pandemic can lead to promotion of remote medical education and democratization of education?

**DOI:** 10.30476/jamp.2020.86090.1217

**Published:** 2020-07

**Authors:** ALIREZA EBRAHIMI, SEDIGHEH EBRAHIMI, SOHEIL ASHKANI ESFAHANI

**Affiliations:** 1 Student research committee, Shiraz University of medical sciences, Shiraz, Iran; 2 Department of medical ethics, Shiraz University of Medical Sciences, Shiraz, Iran; 3 Department of orthopaedic surgery, Massachusetts General Hospital, Harvard Medical School, Boston, Massachusetts, USA

**Dear editor**,

The novel coronavirus (COVID-19) pandemic has been compared to 1918 and 1957 influenza pandemics which took many human lives ( 1 ). The lockdown of cities and educational organizations –which has not been happened since the World War 2- has already disrupted the education at all levels. The United Nations Educational, Scientific and Cultural Organization (UNESCO) has stated more than 1.5 billion students are affected by the current educational disruption ( 2 ). Besides, the current pandemic may have long-term results, as the 1918 pandemic that led to higher rate of physical disability, lower socioeconomic status, decreased income, and reduced education achievement in the adults who were born during the crisis ( 3 ). The devastating impacts of this pandemic on education are undeniable and both governments and private sectors have to increase their efforts to mitigate this impact, particularly through remote learning.

Medical education is not a different kettle of fish and it has been affected by the spread of COVID-19 at both pre-clerkship and clerkship stages ( 4 ). At the pre-clerkship period, social distancing protocols have stopped students’ participation in group interactions, workshop sessions, and patient care environment. Cancellation of routine appointments and surgical procedures, and lack of personal protective equipment and COVID-19 test kits have made the Association of American Medical Colleges (AAMC) suggest medical schools to pause clinical rotations and propose voluntary participation in patients’ care ( 5 ). Medical schools may not abide AAMC guidelines because of the geographical differences and the shortage of health-care workforce. Prior to current pandemic, remote medical education and online problem-based learning were used during the severe acute respiratory syndrome (SARS) epidemic, and subsequently the electronic (E)-education was evolved in a way that it became a popular method among many faculties ( 4 , 6 ).

In the present global crisis, medical education faculties have substituted online courses for basic and health system sciences. Group interactions and some clinical skills sessions may be held virtually. Moreover, the assessment and evaluation of students are done in online settings. Considering the fact that it is unclear that how long the present situation could persist and the probability of re-occurrence of other pandemics in the future, it is the time that educators should rethink about the current learning systems. 

Today, a great number of countries have announced that they temporarily switch their learning system to E-education for all students. However, we believe that rapid advancing technologies such as online social media, online databases, online multimedia and programs based on artificial intelligence and machine learning could transform the global education system and medical education as well. These technologies have profoundly changed the society and economy, earlier to the current situation ( 7 ). Besides, it has been mentioned that learning through new technologies had been a core theme for many faculties in designing future medical schools ( 8 ). 

The rapidly spreading virus made the education industry to turn to online courses. The role of digital education on democratizing education has been under investigation since a few years ago ( 9 ). Online courses could provide an opportunity for students to learn from the best lecturers in the world. The scholars could also expand their personal network and accelerate their profession development by using online learning. Similarly, this could democratize the medical education as the informative resources will be more equally distributed and lower-benefited physicians could use the same method that is used in more advantaged areas. 

There are challenges that lie ahead of remote medical educations as the medical students gain experience through inpatient and outpatient rotations. To address these problems, designing and performing virtual cases, and involving the students in tele-health system could be advantageous. Forthcoming studies should be continued to utilize and improve the present technological tools to be suited for remote medical education. Besides, the educators should reconsider that in a world that the information is a mouse-click away, the role of technologies could be redefined.

We believe that COVID-19 pandemic with all its distresses is a momentum that can change the digital learning and remote medical education forever. For example, online courses could be used as an alternative for students who cannot attend some classes. Furthermore, the development of digital learning could help the medical students to benefit more from virtual lessons before entering to the clerkship stage. Online medical learning could also help the physicians who work in underprivileged countries to be able to use the same educational resources that are used in the privileged areas. In order to achieve these goals, the health education industry should prepare the necessary electronic infrastructure, and revive its policy regarding the use of digital learning. Moreover, information technology technicians and researchers must try to improve the online tools and increase the online databases.
